# Direct association between pharyngeal viral secretion and host cytokine response in severe pandemic influenza

**DOI:** 10.1186/1471-2334-11-232

**Published:** 2011-08-31

**Authors:** Raquel Almansa, Andres Anton, Paula Ramirez, Ignacio Martin-Loeches, David Banner, Tomás Pumarola, Luoling Xu, Jesús Blanco, Longsi Ran, Guillermo Lopez-Campos, Fernando Martin-Sanchez, Lorenzo Socias, Ana Loza, David Andaluz, Enrique Maravi, Mónica Gordón, Maria C Gallegos, Victoria Fernandez, Cristobal León, Pedro Merino, Maria Ángeles Marcos, Francisco Gandía, Felipe Bobillo, Salvador Resino, Jose Mª Eiros, Carmen Castro, Paula Mateo, Milagros Gonzalez-Rivera, Jordi Rello, Raul Ortiz de Lejarazu, David J Kelvin, Jesus F Bermejo-Martin

**Affiliations:** 1 Infection & Immunity Medical Investigation Unit (IMI). Hospital Clínico Universitario-IECSCYL, Avda Ramón, y Cajal 3, 47005 Valladolid, Spain; 2 Microbiology Service, Hospital Clínic, IDIBAPS, University of Barcelona, Carrer de Casanova 143, 08036, Barcelona, Spain; 3 Critical Care Dep., Hospital Universitario La Fe- SEMICYUC, Valencia, Avda Campanar 21, 46009, Spain; 4 Critical Care Department. Joan XXIII University Hospital, University Rovira i Virgili, IISPV, CIBER, Enfermedades Respiratorias (CIBERes) Tarragona, Spain; 5 University Health Network, Medical Discovery Tower, 3rd floor Room 913-916,101 Collegue Street, Toronto, ON, M5G 1L7, Canada; 6 Critical Care Department, Hospital Universitario Rio Hortega-SACYL- SEMICYUC& CIBER de Enfermedades, Respiratorias (Instituto de Salud Carlos III). C/Dulzaina N° 2 47012 Valladolid, Spain; 7 Medical Bioinformatics Department. Instituto de Salud Carlos III. Ctra.Majadahonda-Pozuelo Km. 2200, Majadahonda, Madrid, Spain; 8 Health & Biomedical Informatics Research, University of Melbourne ABN: 84 002 705 224, CRICOS Provider Number: 00116K, Australia; 9 Critical Care Dep., Hospital Son Llatzer- SEMICYUC, Ctra. Manacor, km 4, 07198 Palma de Mallorca, Spain; 10 Critical Care Department, Hospital N Sra de Valme- SEMICYUC, Carretera Madrid-Cadiz (Pol. Ind. La Palmera), KM 548 41014 Sevilla, Spain; 11 Critical Care Dep., Hospital Clínico Universitario-SACYL/SEMICYUC. Avda Ramón y Cajal 3, 47005, Valladolid, Spain; 12 Critical Care Dep., Hospital Virgen del Camino- SEMICYUC, C/DE IRUNLARREA 4, 31008 Pamplona, Spain; 13 Microbiology Service, Hospital Son Llatzer, Ctra. Manacor, km 4, 07198 Palma de Mallorca, Spain; 14 Laboratory of Molecular Epidemiology of Infectious Diseases, National Centre of Microbiology, Instituto de, Salud Carlos III, Ctra.Majadahonda-Pozuelo Km. 2200 Majadahonda, Madrid, Spain; 15 Microbiology & Immunology Service, Hospital Clínico Universitario-SACYL, Avda Ramón y Cajal 3, 47005, Valladolid, Spain; 16 Microbiology Service. Hospital N Sra de Valme, Carretera Madrid-Cadiz (Pol. Ind. La Palmera), KM 548 41014, Sevilla, Spain; 17 Sequencing Unit. Hospital General Universitario Gregorio Marañón, Dr Esquerdo, Madrid 28007, Spain; 18 Critical Care Dep., area General. Hospital Vall d'Hebron. Institut de Recerca Vall d' Hebron-UAB. CIBERES- SEMICYUC. Paseo Vall d'Hebron, 119-129, 08035, Barcelona, Spain

**Keywords:** cytokines, critical, influenza, patients, virus

## Abstract

**Background:**

Severe disease caused by 2009 pandemic influenza A/H1N1virus is characterized by the presence of hypercytokinemia. The origin of the exacerbated cytokine response is unclear. As observed previously, uncontrolled influenza virus replication could strongly influence cytokine production. The objective of the present study was to evaluate the relationship between host cytokine responses and viral levels in pandemic influenza critically ill patients.

**Methods:**

Twenty three patients admitted to the ICU with primary viral pneumonia were included in this study. A quantitative PCR based method targeting the M1 influenza gene was developed to quantify pharyngeal viral load. In addition, by using a multiplex based assay, we systematically evaluated host cytokine responses to the viral infection at admission to the ICU. Correlation studies between cytokine levels and viral load were done by calculating the Spearman correlation coefficient.

**Results:**

Fifteen patients needed of intubation and ventilation, while eight did not need of mechanical ventilation during ICU hospitalization. Viral load in pharyngeal swabs was 300 fold higher in the group of patients with the worst respiratory condition at admission to the ICU. Pharyngeal viral load directly correlated with plasma levels of the pro-inflammatory cytokines IL-6, IL-12p70, IFN-γ, the chemotactic factors MIP-1β, GM-CSF, the angiogenic mediator VEGF and also of the immuno-modulatory cytokine IL-1ra (p < 0.05). Correlation studies demonstrated also the existence of a significant positive association between the levels of these mediators, evidencing that they are simultaneously regulated in response to the virus.

**Conclusions:**

Severe respiratory disease caused by the 2009 pandemic influenza virus is characterized by the existence of a direct association between viral replication and host cytokine response, revealing a potential pathogenic link with the severe disease caused by other influenza subtypes such as H5N1.

## Background

Two years later, the pathogenesis of the illness caused by 2009 pandemic influenza is still poorly known. Pregnancy, severe obesity, cardiovascular disease and diabetes have been reported as risk factors for critical illness following infection with the pandemic virus [1,2]. Persistence of viral secretion has been found in the patients with the worst outcomes [3-5]. Prompt antiviral treatment has been recognized as a protective factor [6]. A number of studies report increased and persistent levels of cytokines and chemokines paralleling severe disease [7,3,8]. A exacerbated cytokine response can contribute to immune-mediated tissue damage during viral infections [9,10]. In this sense, unregulated cytokine and interferon responses have been described to parallel persistence of viral replication and severe disease in SARS [11]. The cause of the observed hypercytokinemia in severe 2009 pandemic influenza is unclear. As previously described, uncontrolled influenza virus replication could strongly influence cytokine production [5,12,13].

The objective of the present study was to evaluate the relationship between host cytokine responses and pharyngeal viral load in pandemic influenza critically ill patients at admission to the ICU.

## Methods

### Patients

Twenty three patients attending the participants' ICUs with primary viral pneumonia during the acute phase of influenza virus illness with negative respiratory and blood bacterial cultures at admission were recruited from November 1^st ^to December 31^st ^2009. Only those patients with confirmed H1N1 infection by real-time polymerase chain reaction (PCR) were included in the study. Blood samples for plasma and serum were collected by using serum, ethylenediaminetetraacetic acid (EDTA) venous blood vacuum collection tubes following the manufacturer's instructions in the next 24 hours following admission to the ICU, according to a unified protocol for all the participant centers. A pharyngeal sample was collected in parallel. Fifteen healthy volunteers of similar age to the patients were recruited among workers of the University of Valladolid, Spain. A standard survey was employed to collect the clinical data, including history and physical examination, oximetric measurement, haematological, biochemical, radiological and microbiological investigation in all the participant centers. Treatment decisions for all patients were not standardized and were decided by the attending physician. Informed consent was obtained directly from each patient or their legal representative and also from the healthy controls before enrolment. Patient and control identification remained anonymous. Approval of the study protocol in both the scientific and the ethical aspects was obtained from the Scientific Committees for Clinical Research of each one of the participant centers. Samples were stored at -80°C until viral load quantification, cytokine and antibody profiling. Patients were split into two groups, depending on the severity of the respiratory failure. The MV group underwent intubation and ventilation (n = 15); the NMV group was composed of eight patients not requiring mechanical ventilation during ICU hospitalization.

### Microbiology

Viral diagnosis was performed on RNA from pharyngeal swabs in the Microbiology Services of the participant hospitals by reverse transcription-polymerase chain reaction (RT-PCR)-based methods using reagents provided free of charge by the Centers for Disease Control (CDC, Atlanta, GA, USA) or purchased from Roche (Basel, Switzerland) (H1N1 detection set). These samples were also assessed by multiplex PCR (Luminex) with the xTAG RVP kit from Luminex-Abbott for co-infection with respiratory syncytial virus, influenza B virus, parainfluenza viruses 1-4, human metapneumovirus, enteroviruses, rhinovirus, adenovirus, bocavirus and coronaviruses NL63, HKU1, 229E, OC43, in accordance with the manufacturer's instructions. Viral load was quantified in pharyngeal swabs and plasma in the Virology Lab of the WHO associated center at Hospital Clinic in Barcelona, Spain. A first single real time RT-PCR was performed for influenza A viral load quantification with a set of primers and probes specific for the influenza virus matrix protein (M1) gene [14]. A standard graph was constructed from the threshold cycle values obtained from serially diluted cRNA control. cRNA control was manufactured from PCR product cloned into plasmid (pGEM-T Easy Vector System, Promega). This plasmid contained a T7 RNA polymerase promoter upstream of the insert to drive transcription by this enzyme. Previous to be in vitro transcribed using MEGAscript Kit (Ambion), the plasmid was linearized with a restriction enzyme (SpeI, New England Biolabs) downstream of the insert. With an additional DNase treatment any residual DNA was removed from RNA transcript. Finally RNA was purified using MEGAclear Kit (Ambion). The concentration and purity of the RNA control was calculated by measuring OD260/280. Known RNA control was serially diluted ten-fold in sterile water for use in real time PCR. The limit of detection was 1.86 log10 RNA copies/reaction with a 98.3% efficiency (data not shown). Oseltamivir resistance was directly detected in the initial positive pharyngeal swab by RT-PCR and sequencing of a 1296-bp fragment of the neuraminidase gene for the presence of the mutation H274Y by using an ABI 3130XL Genetic Analyzer. Standard respiratory and blood cultures were performed to assess the presence of bacterial and fungal infection [15], along with detection of urinary antigen test to *Legionella pneumophila or Streptococcus pneumoniae*.

### Hemagglutination inhibition assays (HAI)

HAI assays were performed on a 100-μl aliquot of the samples at University Health Network (UHN), Toronto, Ontario, Canada. as previously described [16] using inactivated pandemic influenza A/California/07/2009 antigens [17].

### Immune mediator profiling

Immune mediator levels in plasma were measured in patients and controls by using the multiplex Bio-Rad 27-plex assay (Hercules, CA, USA) at the Infection & Immunity Medical Investigation Unit (IMI), HCUV-IECSCYL, Valladolid, Spain. This system allows for quantitative measurement of 27 different chemokines, cytokines, growth factors and immune mediators while consuming a small amount of biological material. A number of additional soluble mediators were measured by using enzyme-linked inmunosorbent assays (ELISAs): IFN-α (Verikine kits purchased from Pbl Interferon Source, Piscataway, NJ, USA), IL-23, TGF-b1 (Quantikine kits purchased from R&D Systems, Minneapolis, MN, USA), IL28A (Legend Max kit purchased from BioLegend, San Diego, CA, USA). Immune mediator's concentration of each individual sample was normalized against the median of the concentration of the control group (n = 15), and the resultant ratios were compared between groups of patients.

### Statistical analysis

The Mann-Whitney U test was employed for cytokine comparison purposes, since the Saphiro Wilk test evidenced absence of normal distribution of the data, and the Levene test demonstrated absence of homogeneity of variance in both MV and NMV groups. Correlation studies between cytokine levels, viral load and clinical parameters were done by calculating the Spearman correlation coefficients. Fisher's exact test was employed for comparing proportion of fatal cases between patients receiving or not steroids. All statistical tests were two-sided, and P < 0.05 was considered significant.

## Results

### Clinical data (table 1)

Chronic respiratory disease and smoking were the most frequent accompanying medical conditions in our cohort. Three women were pregnant (one of them was undergoing the second trimester of pregnancy and the other two the third one). Patients were admitted to ICU five days in average following the onset of the symptoms (both groups). Patients in the MV group showed the most severe respiratory status at admission, based on their Pa O2/Fi O2 values and SOFA scores. The appearance of bilateral infiltrates in chest X- Rays was a common finding in both groups (MV and NMV). Our patients showed elevated levels of AST and CPK at the first contact with the critical care services. Lymphopenia was observed in all but three MV patients and one NMV. All patients received antiviral treatment (oseltamivir) upon admission to the ICU. Six out of fifteen MV patients (40.0%) received steroidal treatment in the first 24 hours following admission to the ICU for six out of eight patients in the NMV group (75%). As already mentioned, patients did not show clinical or microbiological signs of bacterial infection at admission to the ICU. Bacterial super infection during the hospitalization period was observed in four out of fifteen (26%) MV patients. No patients in the NMV group suffered from bacterial infection during hospitalization. Based on Hemagglutination Inhibition Assay (HAI), only four patients (two MV and two NMV) had developed specific antibodies against pandemic influenza at the time of admission to the ICU. MV patients stayed longer at the ICU than NMV patients. Seven MV patients finally died, while all NMV patients survived. No differences were found in the proportion of fatal cases between the patients who received early treatment with steroids (n = 2 fatal cases) and those who did not receive that treatment (n = 5 fatal cases), *p *> 0.05.

**Table 1:** Clinical characteristics of ventilated and non ventilated patients

	MV (n = 15)	NMV (n = 8)
**Gender (M/F)**	8/7	1/7

**Age**	47.00 (15.55)	37.50 (15.12)

**Pandemic influenza vaccine (yes/no)**	0/15	0/8

**Chronic respiratory disease (yes/no)**	3/12	2/6

**Renal disease (yes/no)**	1/14	2/6

**Cardiovascular disease (yes/no)**	2/13	0/8

**Neurological disease (yes/no)**	3/12	1/7

**Cancer (yes/no)**	0/15	1/7

**Obesity (BMI > 30) (yes/no)**	1/14	1/7

**Diabetes (yes/no)**	2/13	0/8

**Pregnancy (yes/no)**	1/14	2/6

**Dyslipemia (yes/no)**	2/13	0/8

**Alcoholism (yes/no)**	0/15	2/6

**Smoker (yes/no)**	9/6	4/4

**Fatal outcome/survivors**	7/8	0/8

**Duration of symptoms at ICU admission**	4.9 (3.6)	5.0 (2.2)

**Pa O2/Fi O2**	120.9 (55.5)	153.8 (69.7)

**Days at hospital**	20.9 (26.2)	4.5 (1.9)

**Days since onset to intubation**	5.1 (3.7)	n.a

**Oseltamivir**	15/0	8/0

**Duration of symptoms before oseltamivir**	4.8 (3.7)	4.6 (2.4)

**Bilateral infiltrates in chest X-Ray at admission to ICU (yes/no)**	14/1	6/2

**Bacterial overinfection during hospitalization (yes/no)** **(resp culture-RC; hemoculture-HC)**	4/11 *S. marcescens *RC, HC *S. aureus *RC *P. aeruginosa *RC *A. baumanii *RC	0/8

**Presence of specific antibodies against 2009 pandemic influenza at admission to ICU (titre > 1/40) (yes/no)**	2/13	2/6

**SOFA**	6.4 (2.8)	4.0 (1.0)

**Creatinine (ref value: 0.5 - 1.2 mg/dl)**	0.8 (0.4)	1.9 (3.0)

**AST (ref value: 2-38 U/l)**	91.2 (65.3)	73.8 (39.9)

**ALT (ref value: 2-41 U/l)**	43.1 (31.0)	75.6 (59.9)

**CPK (ref value: 24- 194 U/ml)**	850.7 (827.0)	449.0 (697.8)

**Leucocytes (4500-10000/mm**^**3**^)	6277.8 (3189.6)	7258.7 (3973.6)

**Neutrophils (1900-8000/mm**^**3**^)	5431.0 (2888.6)	6268.6 (3771.4)

**Lymphocytes (900-5200/mm**^**3**^)	691.5 (445.6)	636.5 (340.8)

Gender: M: male, F: female. Obesity is considered as Body Mass Index, BMI > 30. PaO2 = pressure of oxygen in arterial blood; FiO2 = fraction of inspired oxygen. Bacterial culture notation: R.C: respiratory culture; HC: hemoculture. SOFA: Sequential Organ Failure Assessment score. AST: Aspartate transaminase. ALT: Alanine transaminase. CPK = creatine phosphokinase. Continuous variables are expressed as mean, (standard deviation)

### Virology

**Figure 1:**
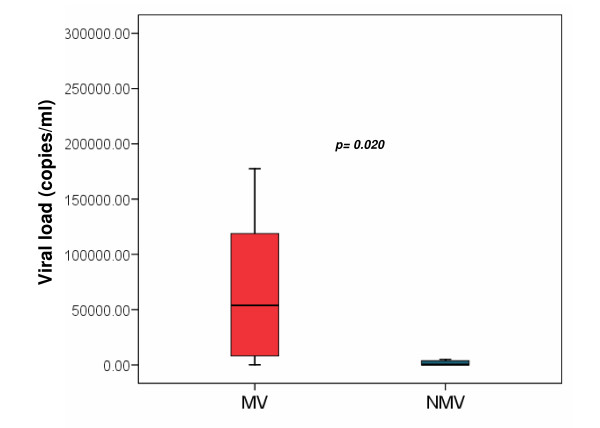
**Box plot representation of pharyngeal viral load in ventilated (MV) and non ventilated patients (NMV)**.

Co-infection by other respiratory viruses was excluded in all the recruited patients. Viral load in pharyngeal swabs (copies/ml) was 300 fold higher in the group of patients with the worst clinical condition (MV) at admission to the ICU (Figure 1). [Median, inter-quartile range]: MV [53925, 129143.75]; NMV [176.81, 4340.47]. When pharyngeal viral load was compared between those patients who received steroids in the first 24 hours following admission to the ICU and those who did not, absence of significant differences between both groups of patients was found. Almost half (7/15) MV patients showed detectable virus in plasma for only one of the NMV patients. Individual values of viral load in plasma in the MV group (copies/ml) were: (1467.5), (348.7); (1423.7); (1656.2); (1276.2); (930.0); (982.5), the last three values corresponding to three patients who eventually died. The NMV patient with detectable virus in plasma showed a viral load of 236.25 copies/ml. We found no significant correlation between viral RNA load in plasma and APACHE II severity score at admission. Mutation H275Y in the neuraminidase gene (NA) was found in one sample collected from a MV male patient who had received oseltamivir (150 mg/24 h) for three days before ICU admission. This patient finally died ten days after the onset of the symptoms and six days after admission to the ICU. Partial NA sequence was uploaded to GenBank database with Accession Number CY064798. The haemagglutinin (HA) mutations D222G/D222N were detected in viral samples obtained from five out of fifteen of the most severe patients: three of these cases showed the D222G variation (all of them showed detectable virus in plasma and one finally died) while two fatal cases showed the D222N mutation (one of them showed detectable virus in plasma). In the NMV group, only one patient showed the D222G variation. MV patients with the viral mutation D222G/N did not show differences in pharyngeal viral load compared to those MV patients for whom this mutation was absent..

### Immune mediator profiling and correlation with viral load

**Figure 2:**
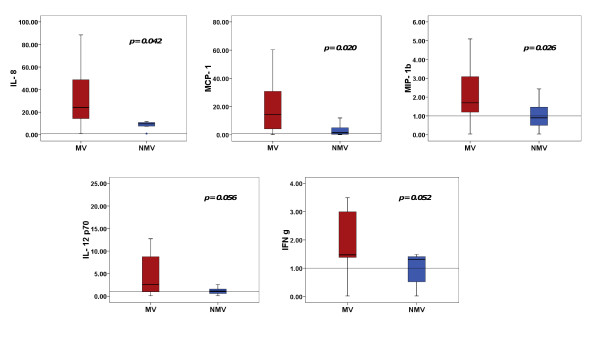
**Comparison of cytokine levels in plasma in ventilated (MV) and non ventilated patients (NMV) by box plots**. Normalized levels against control median are showed.

**Figure 3:**
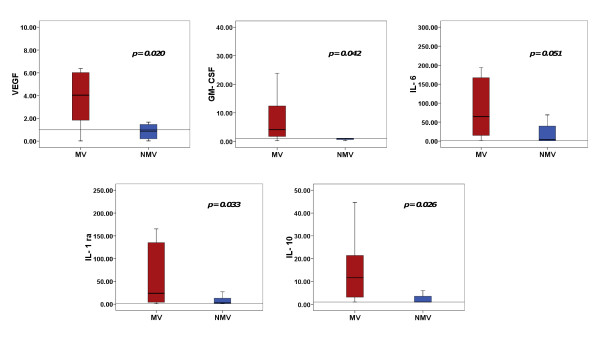
**Comparison of cytokine levels in plasma in ventilated (MV) and non ventilated patients (NMV) by box plots**. Normalized levels against control median are showed.

Comparison of cytokine profiles showed significantly higher levels of three chemokines (IL-8, MCP-1, MIP-1β), two cellular growth factors (VEGF, GM-CSF) and two immuno-modulatory cytokines (IL-1ra, IL-10) in the MV group compared to the NMV one (Figure 2 and 3). In addition, MV patients showed also higher levels of two Th1 cytokines (IL-12p70, IFN-γ) and a pro-Th17 cytokine (IL-6) (*p *< 0.1), When MV and NMV patients were considered as one group, the most important increases as compared to healthy controls corresponded to (mediator, median fold change compared to control): IL-1RA (6.3), IP-10 CXCL10 (23.7), IL-6 (22.7), IL-8 (15.5), G-CSF (5.5), MCP-1 (7.5), IL-10 (5.6) and TGF-β (37.3). On the contrary, IFN-α, IFN-λ (IL-28) and IL-23 were undetectable in the vast majority of the patients in both groups. Comparison of cytokine levels between patients receiving/not receiving steroids in the first 24 hours following admission to the ICU revealed significant higher levels in the latter of IL-1RA, IP-10, VEGF, IL-6, G-CSF, MCP-1, MIP-1β, IL-12p70 and IFN-γ (data not shown).

**Figure 4:**
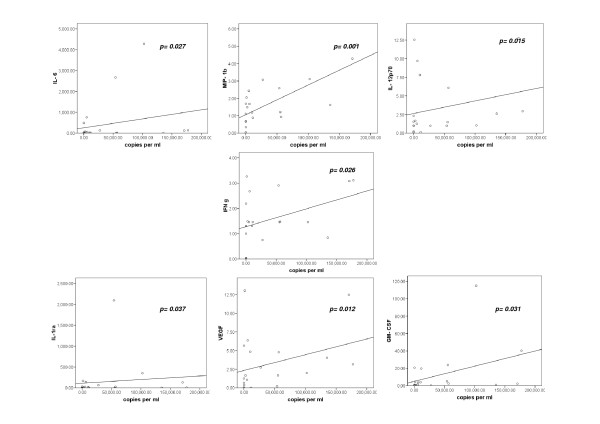
**Correlations between viral load and cytokines (p < 0.05) (n = 23 patients)**.

Spearman test revealed significant positive associations between the following cytokines and viral load [cytokine, (r coefficient)]: IL-1ra (0.44), VEGF (0.52), IL-6 (0.46), MIP-1β (0.65), IL-12p70 (0.50), GM-CSF (0.45), IFN-γ (0.46) (p < 0.05) (Figure 4). Correlation analysis also demonstrated an association between IL-8 (0.37), MCP-1 (0.39) and viral load at the level (p < 0.1). When associations between plasma levels of IL-1ra, VEGF, IL-6, MIP-1β, IL-12p70, GM-CSF, IFN-γ, IL-8 and MCP-1 were examined, a significant positive correlation was found between all of them, except for the pair formed by (IL-8, IL12p70) (data not shown).

## Discussion

The relationship between influenza virus and early host immune response could determine further clinical outcome. In the present study, patients with severe respiratory disease caused by the 2009 pandemic influenza virus showed high levels of viral secretion by the upper respiratory tract at admission to the ICU, evidencing a poor control of viral replication from the very first moments in the course of the disease. These results are in consonance with previous reports showing more active and prolonged viral replication in severe cases of both seasonal and pandemic influenza [18,5].

The observed absence of specific antibodies against the pandemic virus in the vast majority of the patients studied could explain the fail in controlling viral replication. Lack of protective antibodies against the new virus indicates that most patients were still undergoing the innate phase of the host response to the virus at the moment of sampling. In this sense, it is important to highlight that our cohort was constituted by non vaccinated individuals. This observation somehow remarks the potential importance of vaccination for the prevention of severe influenza disease.

Ten patients of this study pertained to a previous cohort for which cytokine were profiled along the period of time comprised from disease onset to the moment of appearance of specific antibodies against the virus [17]. The low number of patients available for inclusion in that period (8 samples from 4 MV patients and 7 samples from 6 NMV patients) precluded perhaps to obtain significant differences between patients with different degree of respiratory involvement in that occasion. Nonetheless, we consequently found in ventilated patients higher levels of the same mediators described in the present article. In this new work we studied a larger cohort of patients (n = 23), and cytokine profiles were compared at a punctual moment in the disease course (admission to the ICU). This approach evidenced that, at admission, the most severe patients (MV) showed an exuberant cytokine and chemokine response, in consonance with previous reports [3,7,8]. In turn, this new work aimed to analyze the relationship between viral replication and host cytokine response. In this sense, the most striking finding was that, as occurs in human cases of influenza A/H5N1 infection [13], 2009 pandemic influenza virus replication is directly associated to the host 'cytokine response, pointing to a central role of viral burden in the pathogenesis of severe forms of this disease.

Correlation studies revealed also that pro and anti inflammatory cytokines are simultaneously regulated in response to the virus. MIP-1β and GM-CSF are chemotactic molecules which mobilize neutrophils, monocytes and macrophages to the site of infection [19]. IL-12p70 and IFN-γ are critical contributors to adaptive immunity, especially in T helper 1 (Th1)-type responses [20,21]. IL-6 has been reported along with IL-12p70 as markers of critical pandemic influenza illness [7,8]. VEGF is a potent angiogenic factor, and is stimulated in hypoxic patients [22]. In addition, it is thought to increase permeability of the bronchial airway epithelium following infection by respiratory viruses [23]. All these mediators are pro-inflammatory, and in consequence, could play a role in the genesis of the respiratory failure in these patients. Alternatively, they could be neccesary to overcome influenza infection in the most severe patients. IL-1ra is an immuno-modulatory molecule. Secretion of IL-1RA could represent an attempt to prevent cytokine-driven inflammatory damage or alternatively to represent a virus-induced evasion mechanism [19,24]. In turn, elevated levels of cytokines such as IL-6 could interfere with the development of an appropriated adaptive immunity response to the virus [25,17,26]. Our results confirm in critically ill patients previous results obtained by Lee et al in hospitalized patients with 2009 pandemic influenza, who demonstrated that high plasma levels of IL-6, IL-8, MCP-1 correlated with the extent and progression of pneumonia [5].

Conditions such as pregnancy, obesity or chronic respiratory diseases could influence cytokine production in response to viral infection [27-29]. Moreover, mechanical ventilation can induce by itself a pro-inflammatory state in the patient [30]. Steroidal treatment could down-modulate cytokine responses also, as in fact seem to occur in our cohort. Nonetheless, a detrimental effect of steroids has been documented in severe pandemic influenza, associated to increased risk of bacterial super-infection in the treated patients [31,32]. On the contrary, steroidal treatment did not impact on pharyngeal viral load at least at the time of sampling in our cohort.

The fact that most patients were not treated or had just begun antiviral treatment at the time of sampling (table 1) could also explain the exacerbated cytokine response observed in our patients. One third of the most severe patients showed the amino acid substitutions D222G/N in the HA of the virus, which is thought to confer an increased predilection of the virus for the lower respiratory tract and has been associated with cases of severe disease [33,34]. Since only one severe patient was infected by a virus containing the H275Y mutation in the NA gene, the potential influence of oseltamivir resistance in the outcome of our patients is limited, though the single patient infected with this mutant virus had a fatal outcome.

## Conclusions

Severe respiratory disease caused by 2009 pandemic influenza virus is characterized by the existence of a direct association between viral replication and host cytokine response, revealing a potential pathogenic link with the severe disease caused by other influenza subtypes such as H5N1.

## List of abbreviations

GM-CSF: granulocyte macrophage colony-stimulating factor; IFN: interferon; IL: interleukin; IL-1RA: interleukin receptor antagonist; IP-10: interferon-gamma inducible protein-10; MCP-1: monocyte chemoattractant protein-1; MIP-1β: macrophage inflammatory protein-1β; MV: mechanical ventilation; NMV: non mechanical ventilation; TGF-b: Transforming growth factor - beta; VEGF: vascular endothelial growth factor.

## Competing interests

The authors declare that they have no competing interests.

## Authors' contributions

AA, TP, MAM implemented the viral load quantification method and sequencing, participated in the data analysis and collaborated writing the manuscript. RA, DK, JFB-M, ROL designed the study, participated in the data analysis and collaborated writing the manuscript. PR, IM-L, JB, LS, AL, DA, EM, MG, CL, PM, FG, FB, PM, JR recruited the patients, designed the clinical survey, collected the clinical information, participated in the data analysis and collaborated writing the manuscript. DB, LX, LR performed the serological assays and participated in writing the manuscript. MCG, VF, JME, CC performed the viral diagnostics and participated in writing the manuscript. GL-C, SR, MG-R provided support with statistical analysis and participated in writing the manuscript. All authors read and approved the final manuscript.

## Authors' information

This work has been developed by an international team pertaining to the Spanish-Canadian Consortium for the Study of Influenza Immunopathogenesis. This Consortium has a large experience in emerging infectious diseases researching, and has contributed to the literature with major articles on the immune response to SARS Coronavirus, H5N1 infection and Pandemic influenza.

## Pre-publication history

The pre-publication history for this paper can be accessed here:

http://www.biomedcentral.com/1471-2334/11/232/prepub
